# Engineering *Pichia pastoris* for improved NADH regeneration: A novel chassis strain for whole-cell catalysis

**DOI:** 10.3762/bjoc.11.190

**Published:** 2015-09-25

**Authors:** Martina Geier, Christoph Brandner, Gernot A Strohmeier, Mélanie Hall, Franz S Hartner, Anton Glieder

**Affiliations:** 1 Austrian Centre of Industrial Biotechnology (ACIB GmbH), Petersgasse 14, Graz, 8010, Austria; 2 Institute of Organic Chemistry, Graz University of Technology, Stremayrgasse 9, Graz, 8010, Austria; 3 Department of Chemistry, University of Graz, Heinrichstrasse 28, Graz, 8010, Austria; 4 Sandoz GmbH, Biochemiestrasse 10, 6250, Kundl, Austria; 5 Institute of Molecular Biotechnology, Graz University of Technology, Petersgasse 14, Graz, 8010, Austria

**Keywords:** bioreduction, cofactor regeneration, dihydroxyacetone synthase, methanol utilization pathway, whole-cell biotransformation

## Abstract

Many synthetically useful reactions are catalyzed by cofactor-dependent enzymes. As cofactors represent a major cost factor, methods for efficient cofactor regeneration are required especially for large-scale synthetic applications. In order to generate a novel and efficient host chassis for bioreductions, we engineered the methanol utilization pathway of *Pichia pastoris* for improved NADH regeneration. By deleting the genes coding for dihydroxyacetone synthase isoform 1 and 2 (*DAS1* and *DAS2*), NADH regeneration via methanol oxidation (dissimilation) was increased significantly. The resulting *Δdas1 Δdas2* strain performed better in butanediol dehydrogenase (BDH1) based whole-cell conversions. While the BDH1 catalyzed acetoin reduction stopped after 2 h reaching ~50% substrate conversion when performed in the wild type strain, full conversion after 6 h was obtained by employing the knock-out strain. These results suggest that the *P. pastoris Δdas1 Δdas2* strain is capable of supplying the actual biocatalyst with the cofactor over a longer reaction period without the over-expression of an additional cofactor regeneration system. Thus, focusing the intrinsic carbon flux of this methylotrophic yeast on methanol oxidation to CO_2_ represents an efficient and easy-to-use strategy for NADH-dependent whole-cell conversions. At the same time methanol serves as co-solvent, inductor for catalyst and cofactor regeneration pathway expression and source of energy.

## Introduction

Bioreductions represent a sustainable strategy to obtain enantiopure molecules which serve as chiral building blocks for fine chemicals and drugs [1–2]. Such reactions are catalyzed by oxidoreductases, which mainly depend on nicotinamide cofactors as electron donors and acceptors. As these cofactors are required in stoichiometric amounts to drive the reaction to completion and the simple addition of these compounds is not acceptable from an economical point of view, efficient in situ regeneration of the consumed cofactor is required. During the recent years, a broad range of cofactor regeneration systems based on enzymatic, chemical, photochemical or electrochemical processes have been developed [3].

In living (growing) cells, NAD(P)H can be recycled via the cellular metabolism of a co-substrate such as glucose. Alternatively, cofactor regeneration enzymes such as D-glucose-6-phosphate dehydrogenase, formaldehyde dehydrogenase and formate reductase can be co-expressed ensuring cofactor supply also in non-growing, resting cells. In addition to the cofactor supply, the application of whole cells circumvents time-consuming enzyme isolation and purification steps and improves enzyme stabilities during a process [4]. Nowadays, efforts are made to further increase the cofactor availability within cells and to allow the use of alternative and cheaper co-substrates by strain engineering [1,5–7].

**Figure 1:**
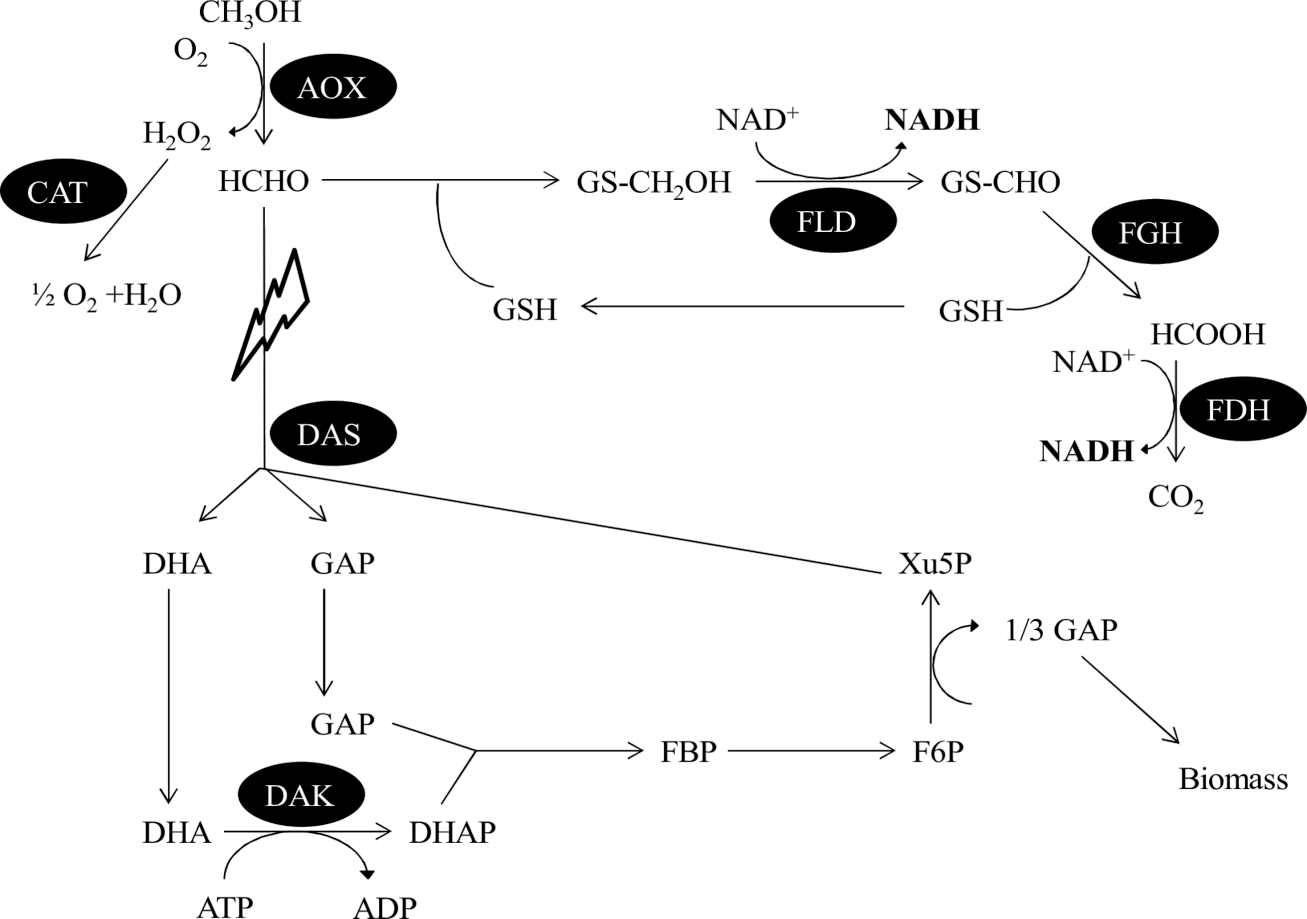
Simplified schematic representation of the methanol utilization pathway in *Pichia pastoris*. The main pathways and the respective enzymes are shown. AOX: alcohol oxidase; FLD: glutathione-dependent formaldehyde dehydrogenase; FGH: *S*-formylglutathione hydrolase; FDH: formate dehydrogenase; CAT: catalase; DAS: dihydroxyacetone synthase; DAK: dihydroxyacetone kinase; DHA: dihydroxyacetone; GAP: D-glyceraldehyde-3-phosphate; DHAP: dihydroxyacetone phosphate; FBP: D-fructose 1,6-bisphosphate; F6P: D-fructose 6-phosphate; Xu5P: D-xylulose 5-phosphate. By deleting dihydroxyacetone synthase the dissimilatory pathway leading to the formation of NADH is strengthened.

Here, we present a novel, engineered platform strain for the use in whole-cell bioreductions and NADH-dependent biosynthetic pathways based on the methylotrophic yeast *Pichia pastoris* (*Komagataella phaffi*). *P. pastoris* is well known as an excellent host for recombinant protein production [8–9]. More recently it also attracted attention as a suitable whole-cell catalyst [10–15]. The target of the presented engineering approach is the methanol utilization (MUT) pathway, which is schematically depicted in Figure 1. This pathway enables methylotrophic yeasts to use methanol as sole carbon source [16]. In the initial step, methanol is oxidized by alcohol oxidase (AOX) to formaldehyde, which is further metabolized either in the assimilatory or in the dissimilatory pathway. In the latter one, formaldehyde spontaneously reacts with glutathione to *S*-hydroxymethylglutathione which is oxidized in a first step by the glutathione- and NAD^+^-dependent enzyme formaldehyde dehydrogenase (FLD) to *S*-formylglutathione. *S*-Formylglutathione hydrolase (FGH) then hydrolyses this compound to formate and glutathione. In a second NAD^+^-dependent step, formate is oxidized to CO_2_ by formate dehydrogenase (FDH). Thus, 2 equivalents of the cofactor NADH are generated via the dissimilatory pathway and full oxidation of the single carbon molecule methanol. Alternatively, in a simplified model formaldehyde is condensed with D-xylulose-5-phosphate and subsequently converted into dihydroxyacetone and D-glyceraldehyde-3-phosphate by dihydroxyacetone synthase (DAS), thereby contributing to biomass production on methanol.

Modifying the dissimilatory part of the MUT pathway has already been shown to improve the substrate conversion by NADH-dependent enzymes in *P. pastoris* [7]*.* Over-expression of the formaldehyde dehydrogenase, which was identified as the main bottleneck for an efficient cofactor recycling via the dissimilatory pathway, significantly improved the production rates of NADH-dependent whole-cell biotransformations. In contrast, the present study focuses on redirecting the flux in the MUT pathway by disrupting the assimilation pathway. Thus, the co-substrate methanol should be exclusively redirected to cofactor regeneration in theory, increasing the available NADH concentration in whole-cell biotransformations, while cells should not be able to grow any more if methanol is the sole carbon source.

## Results and Discussion

### Construction of knock-out strains

The assimilatory pathway was disrupted by the knock-out of dihydroxyacetone synthase. In contrast to other methylotrophic yeasts, *P. pastoris* has two genes encoding two isoforms of this enzyme (*DAS1* and *DAS2*) [17–18]. Therefore, three knock-out strains were generated to investigate their impact on cofactor regeneration: the single knock-outs *Δdas1* and *Δdas2* as well as the double knock-out *Δdas1 Δdas2*.

**Figure 2:**
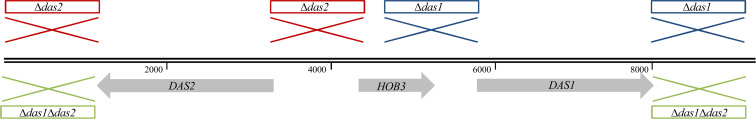
Representation of the genomic region coding for dihydroxyacetone synthases. The *DAS1* and *DAS2* coding sequences are located in close proximity on chromosome 3 in opposite direction. The two genes are separated by a short sequence encoding a hypothetical guanosine nucleotide exchange factor (*HOB3*). The target sites for the respective *das* knock-out cassettes are indicated schematically.

The targeted gene disruption was accomplished with knock-out cassettes as described earlier [19–20]. The cassettes were harboring a Zeocin^TM^ resistance marker for the selection of integrative transformants and the FLP recombinase system [21] to enable marker recycling. Thereby, marker-free knock-out strains were obtained which only have one 34 bp FLP recombination target sequence (FRT) left in the targeted locus. The cassettes were designed such that the complete coding sequence including the start and stop codons of the corresponding *DAS* gene was deleted as schematically depicted in Figure 2. In the case of *Δdas1 Δdas2*, the coding sequences of both genes were deleted in one step, thereby also removing *HOB3*, coding for a hypothetical guanosine nucleotide exchange factor.

### Characterization of knock-out strains

In a first step, the generated *das* knock-out strains were characterized by determining their specific growth rate on different carbon sources. As expected, the growth of the single and double knock-out strains on D-glucose and glycerol was not significantly impaired, showing growth rates in the same order of magnitude as the wild type strain (Table 1). A distinct phenotype was observed when methanol was used as the sole carbon source for growth. A slight, but residual growth was observed for the double knock-out strain, although both dihydroxyacetone synthase genes, that are linking methanol to biomass production, were deleted. In the course of 38 h, the optical density of the *P. pastoris Δdas1 Δdas2* cultures on methanol doubled. This remaining carbon flux into cellular metabolism might also enable the continuation of protein expression and thus biocatalyst production, while still maintaining a strong methanol flux towards oxidation to CO_2_ and NADH regeneration.

**Table 1:** Specific growth rates of *P. pastoris* CBS7435 and generated *das* knock-out strains on different carbon sources. Values represent mean values ± standard deviations of the growth rate during the exponential growth phase determined in biological triplicates.

Strain	Growth rate [h^−1^]		
	D-Glucose	Glycerol	Methanol

*P. pastoris* CBS7435	0.25 ± 0.03	0.19 ± 0.05	0.15 ± 0.03
*P. pastoris* CBS7435 *Δdas1*	0.23 ± 0.06	0.21 ± 0.04	0.12 ± 0.03
*P. pastoris* CBS7435 *Δdas2*	0.23 ± 0.03	0.20 ± 0.02	0.09 ± 0.01
*P. pastoris* CBS7435 *Δdas1 Δdas2*	0.26 ± 0.03	0.20 ± 0.04	0.01 ± 4 × 10^−3^

Deleting only one isoform of the dihydroxyacetone synthase did not have such a severe impact on the methanol depending growth of *P. pastoris*. The growth rates were reduced by ~20% and by ~40% for the *Δdas1* and *Δdas2* single knock-out strains, respectively.

The behavior of the *das* knock-out strains in heterologous protein production was tested by expressing green fluorescent protein (GFP) under the control of the *AOX1* promoter (P*_AOX1_*), which is the most prominent, methanol inducible promoter for *P. pastoris*.

**Figure 3:**
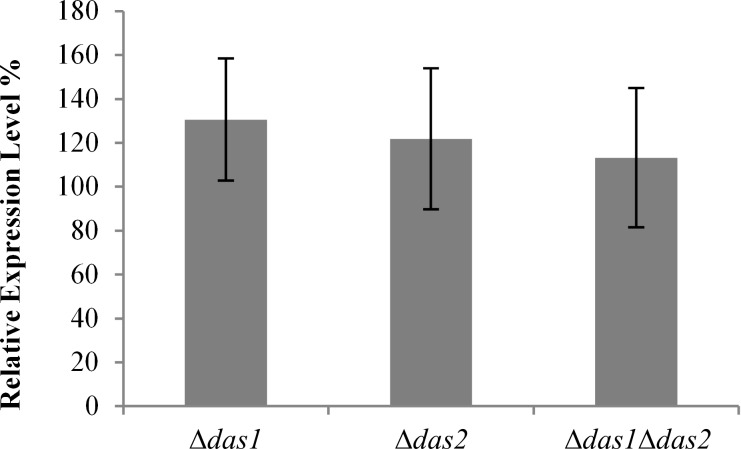
Relative expression levels of the green fluorescent protein (GFP) in the *das* knock-out strains. GFP expression obtained in the wild type *P. pastoris* strain indicated as relative fluorescence units per OD_600_ unit was set as 100%. GFP fluorescence was measured after 70 h of methanol induction. The shown values represent mean values ± standard deviations of 40 individual transformants.

A possible negative effect on P*_AOX1_* driven protein expression might arise from changes in the energy metabolism due to the interrupted input into the pentose phosphate cycle. In addition, a possible accumulation of methanol oxidation products due to the lack of the two key metabolizing enzymes might be unfavorable for recombinant protein production. However, as shown in Figure 3, the P*_AOX1_* driven expression of GFP was not negatively affected by deleting the *DAS* gene(s). In fact, the obtained expression levels in the generated single knock-out strains were increased up to ~30% in comparison to the wild type *P. pastoris* strain. These findings are of importance for the use of the knock-out strains in whole-cell processes, as impairments in recombinant protein production, i.e., the production of the actual biocatalyst, would reduce its applicability.

### NADH-dependent biotransformations performed in knock-out strains

To evaluate the NADH regeneration potential of the generated knock-out strains, they were employed as hosts in whole-cell bioreductions based on the 2,3-butanediol dehydrogenase from *S. cerevisiae* YAL060W (BDH1). This enzyme was shown to specifically use NADH as cofactor, displaying high turnover numbers in the reduction of racemic acetoin (*k*_cat_ = 98,000 min^−1^) [22].

**Figure 4:**
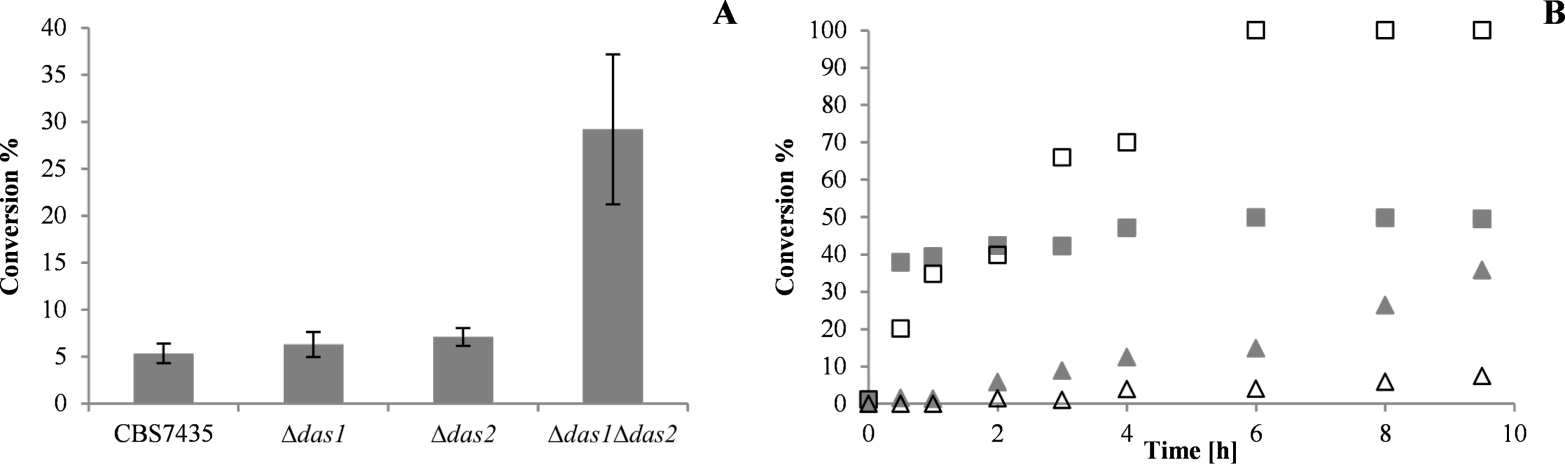
BDH1-based whole-cell conversions of *rac*-acetoin. (A) Conversions of 25 mM *rac*-acetoin in the different knock-out strains were performed in 96 well deep-well plates for 16 h. The shown conversions represent mean values ± standard deviations derived from reactions using whole cells from 40 individual BDH1 transformants. (B) Time-resolved biotransformations of 50 mM substrate were conducted in the wild type (grey squares) and in the *Δdas1 Δdas2* strain background (white squares). Background activity by endogenous acetoin reducing enzymes of *P. pastoris* was observed (grey triangles: *P. pastoris* CSB7435 wild type, white triangles: *P. pastoris Δdas1 Δdas2*).

In an initial screening step, BDH1 transformants in the respective wild type and knock-out backgrounds were evaluated by performing acetoin conversions in the 96 well deep-well format. After biomass production on a D-glucose containing medium, methanol was fed as the sole carbon source to induce the production of the BDH1 enzyme and to induce the dissimilatory pathway. After 12 hours of induction acetoin was added to the growing cells as substrate in addition to the co-substrate methanol. The substrate conversions into the two products (2*R,*3*R*)-butane-2,3-diol and *meso*-butane-2,3-diol obtained after 16 h reaction time are shown in Figure 4A. Performing the BDH1-catalyzed acetoin reduction with the wild type strain *P. pastoris* CBS7435 resulted in an average conversion of ~5% (40 individual clones tested). Employing the single knock-out strains *Δdas1* and *Δdas2* in the same reaction did not yield substantially higher conversions. The average conversions were in the same order of magnitude as for the wild type strain (~6% and ~7% for the *Δdas1* and *Δdas2* strain, respectively), indicating that the NADH regeneration was not significantly improved in these strains. In contrast to the single knock-out strains, the acetoin conversion was 5.5-fold higher when using the double knock-out strain in comparison to the wild type (average conversion ~29%), indicating a more efficient cofactor supply. The higher conversions with the double-knock out strain were even obtained with fewer cells per reaction as it grew much slower on methanol than the wild type and the single knock-out strains. These first findings confirm the assumption that the NADH formation via methanol oxidation is increased when the assimilatory pathway is disrupted, if there is a NADH requirement in the cells due to, e.g., a biocatalytic reaction. The latter is only efficiently realized when both genes coding for the dihydroxyacetone synthase proteins are deleted.

The time course of acetoin conversions with representing BDH1 transformants in the wild type and *Δdas1 Δdas2* background were investigated under optimized conditions in shake flasks. To avoid differences in the amount of cells employed in the biotransformation and to obtain higher product yields, cells corresponding to 3000 OD_600_ units were harvested 12 h after the first methanol induction and resuspended in 50 mL of buffered minimal medium before the conversions were started by the addition of substrate. During the first two hours of the whole-cell biotransformation, the conversions obtained with each of the strains were in the same order of magnitude (see Figure 4B). After longer reaction times, hardly any residual BDH1 activity was observed with the wild type strain (final conversion of ~50%), while the reaction reached 100% conversion after 6 h in the engineered strain. These findings clearly indicate that the NADH supply for the oxidoreductase can be attained over a prolonged reaction time in the *P. pastoris Δdas1 Δdas2* strain, which directly translates into increased yields in the whole-cell reactions.

It has to be noted, that an additional acetoin reduction by endogenous *Pichia* dehydrogenases was also observed for the wild type and the double knock-out strain without the *BDH1* expression cassette, reaching ~35% and ~8% substrate conversion, respectively. As for the recombinant strains, two products were detected, namely (2*R,*3*R*)-butane-2,3-diol and *meso*-butane-2,3-diol.

## Conclusion

Efficient cofactor recycling is not only required for an optimal enzyme performance, but is also crucial to render the resulting process economical and sustainable. For this purpose, biotransformations based on whole cells represent an elegant strategy as the cell metabolism can be exploited for cofactor supply. We have generated a novel platform strain for whole-cell catalysis based on the methylotrophic yeast *P. pastoris* by engineering its methanol utilization pathway. By deleting the pathway for methanol assimilation, we forced flux through the dissimilatory pathway in which theoretically two molecules of NADH can be formed per molecule of methanol. The resulting double knock-out strain *P. pastoris* CBS7435 *Δdas1Δdas2* displayed better performance in dehydrogenase based whole-cell biotransformations without the need of an external electron source. Higher conversions were achieved in comparison to reactions carried out with the wild type *Pichia* strain due to an extended NADH supply by the oxidation of methanol.

Methanol, which is a cheap carbon source, fulfills several roles in the presented biotransformations: It acts as an inducer of the P*_AOX1_* regulated production of the redox enzyme as well as of the endogenous NADH regeneration system at the same time. The cofactor regeneration itself is based on methanol as co-substrate: the oxidation of methanol is irreversible and represents a strong thermodynamic driving force while only CO_2_ is produced as side product. Furthermore, methanol can serve as solvent for the substrate and methylotrophic yeasts are already naturally adapted to elevated concentrations of methanol.

The redesigned *Pichia* strain, thus, represents a valuable host for whole-cell applications where NADH regeneration is an issue. No additional over-expression of cofactor regenerating enzymes such as formate dehydrogenase, which only yields one molecule of NADH per carbon atom, is required. Due to its simplicity it might further boost the use of recombinant whole-cell catalysts in chemical and pharmaceutical industry.

## Experimental

### General

Unless stated otherwise, all chemicals were obtained from Sigma-Aldrich (Steinheim, Germany) or Carl-Roth (Karlsruhe, Germany) with the highest purity available. Zeocin^TM^ was obtained from InvivoGen (San Diego, CA, USA). Phusion^®^ High Fidelity Polymerase for DNA amplification and further DNA modifying enzymes were purchased from Thermo Fisher Scientific Inc. (Waltham, MA, USA). *E. coli* Top10 (Invitrogen, Carlsbad, USA) was used for all cloning steps and plasmid propagation. The *P. pastoris* strain CBS7435 as well as the plasmid pPp_T4_S were obtained from the *Pichia* pool of TU Graz [20].

### Generation of *das* knock-out strains

The generation of knock-out strains was based on linear excision cassettes harboring a FLP recombinase system for inducible marker recycling. A schematic representation of such a knock-out cassette is provided in Supporting Information File 1, Figure S1. Knock-out cassettes were essentially constructed as described in [20]. Locus specific integration sequences, i.e., DNA fragments of approximately 1.5 kb directly located up- and downstream of the corresponding coding sequences, were amplified from genomic DNA. The corresponding primers were designed based on the genome sequence of *P. pastoris* CBS7435 and are summarized in Supporting Information File 1, Table S1. The resulting knock-out cassettes were employed for the transformation of *P. pastoris* CBS7435 wild type cells according to the condensed protocol by Lin-Cereghino et al. [23]. Transformants were selected on YPD agar plates containing 100 mg/L Zeocin^TM^. The correct integration of the knock-out cassette was confirmed by PCR using genomic DNA as template and primer pairs binding within the cassette and either up- or downstream of the targeted locus (see Supporting Information File 1, Table S2). In a next step, the marker was recycled by inducing the FLP recombinase. Therefore, a single colony from a correct knock-out strain was used to inoculate 50 mL of YPD media (250 mL baffled shake flask) and grown for 24 h at 28 °C. After 24 h of growth, methanol was added to a final concentration of 0.5% to start recombinant protein production, which was maintained by daily methanol addition for 96 h. An aliquot of these cultures was plated on YPD agar plates to obtain single colonies. These were subsequently streaked out on YPD agar plates supplemented with 100 mg/L Zeocin^TM^ to identify clones that had excised the cassette and, thus, were Zeocin^TM^-sensitive. The marker recycling was further validated by PCR using primers that bind in the genomic region directly up- and downstream of the deleted locus (see Supporting Information File 1, Table S3) and Sanger sequencing of the resulting PCR product.

### Growth rate studies

Liquid *Pichia* cultures were grown in buffered minimal medium containing 200 mM KP_i_ (pH 6.0), 13.4 g/L yeast nitrogen base and 0.4 mg/L biotin supplemented either with 2% (w/v) D-glucose (BMD), 1% (w/v) glycerol (BMG) or 0.5% (v/v) methanol (BMM). Growth rates of the generated *das* knock-out strains were determined by measuring the optical density (OD_600_) of cultures in biological triplicate during the exponential growth phase.

### GFP expression in knock-out strains

The plasmid pPp_T4_S_GFP was linearized with *Smi*I and used to transform the wild-type as well as the *das* knock-out *P. pastoris* strains. Single colonies were transferred to 96 well deep-well plates for standard cultivation and protein production as described previously [24]. For comparison of the expression levels, the GFP fluorescence (488 nm excitation, 507 nm emission) and the optical density (OD_600_) of the cell suspension were measured with a Synergy MX Microplate Reader.

### Cloning of model enzyme

To evaluate the cofactor regeneration of the generated knock-out strains, the 2,3-butanediol dehydrogenase of *S. cerevisiae* YAL060W (BDH1) [22] was employed as NADH-dependent model enzyme. The corresponding gene was ligated with the *P. pastoris* expression vector pPp_T4_S_after digestion with *EcoR*I/*Not*I. The thus resulting construct, pPp_T4_S_BDH1, was linearized with *Smi*I for the subsequent transformation of the *Pichia* wild-type as well as *das* knock-out strains.

### Screening of BDH1 transformants

Strains harboring the expression cassette for *BDH1* were grown for 60 h in 250 µL of BMD medium in 96 well deep-well plates. Protein expression was started by the addition of 250 µL BMM2 (1% (v/v) in methanol) approximately 12 h prior to the start of the bioreduction reaction. The BDH1-mediated bioreduction was started by adding BMM10 (5% (v/v) methanol) containing 250 mM *rac*-acetoin. After a reaction time of 16 h, 300 µL of the reaction supernatant were extracted twice with 400 µL ethyl acetate containing 50 mM *n*-butanol as internal standard for the determination of acetoin conversions. The combined organic phases were dried over Na_2_SO_4_ and subjected to GC-FID analyses as described below.

### Kinetic studies of biotransformations

Biotransformations in shake flasks were conducted with strains harboring one copy of the *BDH1* expression cassette as determined by quantitative real-time PCR [25]. The corresponding strains were grown for 60 h in 200 mL BMD medium (2 L baffled shake flasks) and protein expression was started by the addition of 20 mL BMM10 approximately 12 h prior to the start of the bioreduction reaction. For BDH1 catalyzed acetoin conversion, cells corresponding to 3000 OD_600_ units were harvested by centrifugation (3000*g*, 10 min, rt) and resuspended in 50 mL of buffered minimal medium. The reaction was started by adding 10 mL of the substrate solution (300 mM *rac*-acetoin, 50% (v/v) methanol, 200 mM KP_i_, pH 6.0).

### GC-FID analysis of biotransformations

Acetoin conversions were determined using an Agilent Technologies 6890N gas chromatograph equipped with an FID detector and a CTC Analytics CombiPAL autosampler. A Chirasil-DEX CB column (25 m × 0.32 mm, 0.25 µm film) was applied using H_2_ as carrier gas. The samples were injected without split. The following temperature program was used: 65 °C for 6.5 min; 50 °C/min to 80 °C; 80 °C for 0.7 min; 2 °C/min to 85 °C; 85 °C for 3 min.

Retention times: *n*-butanol: 1.71 min, (*S*)-acetoin: 2.04 min, (*R*)-acetoin: 2.32 min, (2*R,*3*R*)-butane-2,3-diol: 8.27 min, *meso*-butane-2,3-diol: 8.90 min.

## Supporting Information

File 1 Schematic representation of knock-out cassette architecture and sequences of primers used.
