# Supplementary material for: Engineering Pichia pastoris for improved NADH regeneration: A novel chassis strain for whole-cell catalysis
Source: Beilstein J Org Chem. 2015 Sep 25;11:1741–8. doi: 10.3762/bjoc.11.190 (PMC4660914; doi:10.3762/bjoc.11.190)
Supplement: File 1 — Schematic representation of knock-out cassette architecture and sequences of primers used. [file Beilstein_J_Org_Chem-11-1741-s001.pdf]

Supporting Information

for

Engineering *Pichia pastoris* for improved NADH regeneration: A novel chassis strain for whole-cell catalysis

Martina Geier[1], Christoph Brandner[1], Gernot A. Strohmeier[1,2], Mélanie Hall[3], Franz S. Hartner[4,5] and Anton Glieder[5*]

Address: [1]Austrian Centre of Industrial Biotechnology (ACIB GmbH), Petersgasse 14, Graz, 8010, Austria, [2]Institute of Organic Chemistry, Graz University of Technology, Stremayrgasse 9, Graz, 8010, Austria, [3]Department of Chemistry, University of Graz, Heinrichstrasse 28, Graz, 8010, Austria, [4]current address: Sandoz GmbH, Biochemiestrasse 10, 6250, Kundl, Austria and [5]Institute of Molecular Biotechnology, Graz University of Technology, Petersgasse 14, Graz, 8010, Austria

Email: Anton Glieder[*] - a.glieder@tugraz.at

[*]Corresponding author

Schematic representation of knock-out cassette architecture and sequences of primers used

Generation of das knock-out strains

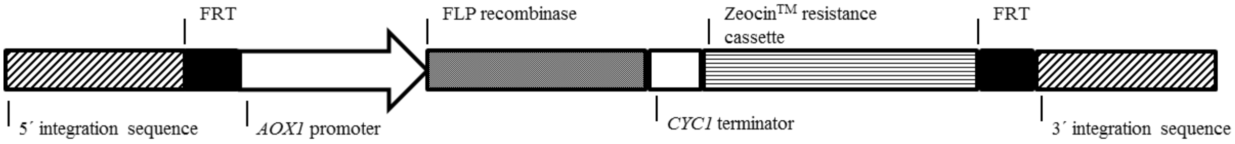

Figure S1: Schematic representation of knock-out cassette architecture. The core is composed of a P_{AOX1} based expression cassette for the FLP recombinase as well as of a Zeocin resistance marker cassette. These parts are surrounded on both sides by recombinase target sequences (FRT) and locus specific integration sequences. Components are not drawn to scale.

Table S1: Sequences of primers used for the amplification of the 5´- and 3´- integration sequences of the *das* knock-out cassettes.

**Primer**	**Sequence (5´- 3´)**
3UTRDAS1F	TCG GCC GAT CAG GCC ACG GGA AGT CTT TAC AGT TTT AGT TAG
3UTRDAS1R	GCA TAT CGT AGT CCA ATT TAA ATT GTC ATA CAG ATC CAA TGC TGC
5UTRDAS1F	ATG ACA ATT TAA ATT GGA CTA CGA TAT GCT CCA ATC C
5UTRDAS1R	TCG GCC CTA GTG GCC GTT GTT TGT AAG TAA ACG AAT CAA GAT ACT G
3UTRDAS2F	TCG GCC GAT CAG GCC TTT TGA TGT TTG ATA GTT TGA TAA GAG TGA AC
3UTRDAS2R	GGA ATA AGC AGA ACT GTA GAT TTA AAT CAA ACT CTT CAT CCA GAC TCT CAT C
5UTRDAS2F	GAA GAG TTT GAT TTA AAT CTA CAG TTC TGC TTA TTC CCC C
5UTRDAS2R	TCG GCC CTA GTG GCC GTA GAT TTG GCC ACT AAC GGG TTA G
3UTRDAS1&2_cleanR	AAG CAG AAC TGT AGA TTT AAA TTG TCA TAC AGA TCC AAT GCT GC
5UTRDAS1&2_cleanF	TTG GAT CTG TAT GAC AAT TTA AAT CTA CAG TTC TGC TTA TTC CCC

Table S2: Sequences of primers used to verify the correct insertion of the corresponding *das* knock-out cassettes.

**Primer**	**Sequence (5´- 3´)**
Up5UTRDAS1F	TACCCAATTCAGTGGAACCGTTC
Down3UTRDAS1R	CTC TGC TAG TAA GGT ACA TCA TCA CGG TC
Up5UTRDAS2F	GAT GTA AGA CGT GAC GAT GAT TGG
Down3UTRDAS2R	TAA TCC GGA AGT TCT TCT CCT GG
PAox1SeqR	GGTTTCATTCAACCTTTCGTCTTTGGATG
PucSeqF	CTTTTTACGGTTCCTGGCCTTTTGC

Table S3: Sequences of primers used for the verification of the *das* knock-out strains after marker recycling.

**Primer**	**Sequence (5´- 3´)**
DAS1_check_fw	GCAGGATGCCTGATATATAAATCCCAGATGATC
DAS1_check_rev	CATCAGATATTATCATCGCGGCTTACGTAATAAC
DAS2_check_fw	CCATCCCACCCTAGGATGTCCTACAGG
DAS2_check_rev	CAAGTTCGTTTTAACTTAAGACCAAAACCAGTTACAAC
